# Robust Innate Immunity of Young Rabbits Mediates Resistance to Rabbit Hemorrhagic Disease Caused by Lagovirus Europaeus GI.1 But Not GI.2

**DOI:** 10.3390/v10090512

**Published:** 2018-09-19

**Authors:** Matthew J. Neave, Robyn N. Hall, Nina Huang, Kenneth A. McColl, Peter Kerr, Marion Hoehn, Jennifer Taylor, Tanja Strive

**Affiliations:** 1 CSIRO Australian Animal Health Laboratory, Geelong, VIC 3220, Australia; Matthew.Neave@csiro.au (M.J.N.); Kenneth.Mccoll@csiro.au (K.A.M.); 2 CSIRO Health and Biosecurity, Canberra, ACT 2601, Australia; Robyn.Hall@csiro.au (R.N.H.); Nina.Huang@csiro.au (N.H.); Peter.Kerr@csiro.au (P.K.); Marion.Hoehn.1206@gmail.com (M.H.); 3 CSIRO Agriculture and Food, Canberra, ACT 2601, Australia; Jen.Taylor@csiro.au

**Keywords:** rabbit, rabbit hemorrhagic disease virus, RHDV, RHDV-2, GI.1, GI.2, RNA-Seq, transcriptome, lagovirus, calicivirus

## Abstract

The rabbit caliciviruses Lagovirus europaeus GI.1 and GI.2 both cause acute necrotizing hepatitis in European rabbits (*Oryctolagus cuniculus*). Whilst GI.2 is highly virulent in both young and adult rabbits, rabbits younger than eight weeks of age are highly resistant to disease caused by GI.1, although they are still permissive to infection and viral replication. To investigate the underlying mechanism(s) of this age related resistance to GI.1, we compared liver transcriptomes of young rabbits infected with GI.1 to those of adult rabbits infected with GI.1 and young rabbits infected with GI.2. Our data suggest that kittens have constitutively heightened innate immune responses compared to adult rabbits, particularly associated with increased expression of major histocompatibility class II molecules and activity of natural killer cells, macrophages, and cholangiocytes. This enables them to respond more rapidly to GI.1 infection than adult rabbits and thus limit virus-induced pathology. In contrast, these responses were not fully developed during GI.2 infection. We speculate that the observed downregulation of multiple genes associated with innate immunity in kittens during GI.2 infection may be due to virally-mediated immunomodulation, permitting fatal disease to develop. Our study provides insight into the fundamental host–pathogen interactions responsible for the differences in age-related susceptibility, which likely plays a critical role in defining the success of GI.2 in outcompeting GI.1 in the field.

## 1. Introduction

Rabbit hemorrhagic disease (RHD) is a peracute and often lethal hepatitis caused by the rabbit caliciviruses *Lagovirus europaeus* GI.1 (previously called RHDV) and *Lagovirus europaeus* GI.2 (previously called RHDV2 or RHDVb) [1,2,3]. Both viruses are positive-sense RNA viruses belonging to the genus *Lagovirus*, family *Caliciviridae*. GI.1 was first reported in China in 1984, and spread rapidly through European rabbit (*Oryctolagus cuniculus*) populations in Asia and Europe, with outbreaks also reported from North Africa and the Americas in the 1980s [1]. GI.1 was released in Australia in the mid-1990s to control wild rabbit populations, and spread to New Zealand in 1997 [4]. It is now considered endemic in wild rabbit populations in Asia, Europe, Australia, New Zealand, and parts of Africa [1]. Subsequently, a novel lagovirus, GI.2, emerged in France in 2010 [2]. GI.2 has since been detected throughout Europe and nearby islands [2,5,6,7,8,9,10,11,12], and in Australia in 2015 [13]. GI.2 is now endemic in Europe and Australia, and appears to be replacing GI.1 strains in these regions [5,10,14,15].

GI.2 is antigenically and genetically distinct from GI.1, and is able to cause disease in GI.1-vaccinated rabbits and in various hare species, including Italian hares (*Lepus corsicanus*), Sardinian cape hares (*Lepus capensis mediterraneus*), and European brown hares (*Lepus europaeus*) [2,5,16,17,18,19,20]. Both viruses have an identical genome organisation comprising two open reading frames (ORFs). ORF1 encodes seven non-structural proteins and the major capsid protein VP60, while ORF2 encodes a minor structural protein, VP10 [1]. Interestingly, multiple GI.2 recombinants have been reported, containing non-structural gene sequences of either GI.2, GI.1, or GI.4 (a group largely comprised of non-pathogenic lagoviruses) with a recombination breakpoint located at the junction between the non-structural genes and VP60 [15,21]. All of these recombinants have been reported to be virulent in rabbits as well as hares [22]. This suggests that determinants of pathogenicity, as well as the broader host range of GI.2 viruses, lie within the structural gene sequences.

In natural infections, the virus usually enters the host via mucosal membranes after ingestion [23]. However, experimentally, animals can also be infected through parenteral inoculation. Orally-infected rabbits showed an increased survival time compared to animals infected by intramuscular or intradermal injection; however, it is not known whether this is due to a prolonged disease duration or to delayed virus entry in orally-infected animals [24]. Typically, over 90% of adult rabbits infected with GI.1 develop pyrexia within 24 to 36 h post infection (hpi) and die 12 to 36 h later [1]. In contrast, disease associated with early (2010 to 2011) strains of GI.2 was frequently subacute, with mortalities occurring three to nine days post-infection, and highly variable case fatality rates between experiments [16]. Pathogenicity studies on more recent GI.2 strains (2014 to 2015) showed case fatality rates comparable with GI.1, although low numbers of animals were tested [25]. Typical necropsy lesions associated with both GI.1 and GI.2 viruses include extensive hepatic necrosis, initially with a predominantly periportal distribution, splenomegaly, and hemorrhages throughout most solid organs resulting from disseminated intravascular coagulation (DIC; reviewed in Reference [1]).

Studies on lagovirus replication have thus far been impeded by the lack of a reliable cell culture system, and in vivo studies on pathogenicity mechanisms have largely been limited to GI.1. The pathogenesis of GI.2 is presumed to follow a similar pattern to that of GI.1, although the chronology of infection may differ. Cellular receptors have not yet been identified for either virus, although histo-blood group antigens (HBGAs), complex carbohydrates linked to glycoproteins or glycolipids that are expressed on epithelial cells of the trachea and duodenum in rabbits, have been identified as co-receptors/attachment factors for lagoviruses [26,27,28,29,30,31]. Viral replication is presumed to occur predominantly in hepatocytes, and GI.1 antigen can be detected in these cells as early as 12 hpi [32,33]. Viral antigens have also been detected in cells of the monocyte/macrophage lineage, including Kupffer cells and intravascular macrophages, most commonly in the lungs and spleen [33,34,35,36,37,38,39]. Infected hepatocytes initially show hydropic change and ultimately become apoptotic and necrotic [34,37,40,41,42]. Necrotic foci are associated with infiltration of inflammatory cells, predominantly heterophils [18,37,41,43]. Necrotic foci coalesce as infection proceeds and this widespread hepatic damage leads to typical biochemical changes associated with hepatic disease, including marked elevations in serum transaminases and bilirubin [43,44]. Subsequently, infected rabbits develop DIC, characterized by thrombocytopenia and prolongations in blood clotting times [44,45,46].

In contrast to the severe pathology induced in adult rabbits, young rabbits are innately resistant to disease caused by GI.1, although they can be infected and seroconvert, even after the inoculation of low infectious doses [47,48,49]. This resistance to disease is independent of maternal antibody status and is gradually lost over time, with case-fatality rates increasing in a non-linear fashion until approximately nine weeks of age, when case-fatality rates are comparable to those for adult rabbits [23,50]. The viral antigen can only be detected in a limited number (0.01 to 0.2%) of hepatocytes in young rabbits, compared to over 60% of hepatocytes in adult animals [33,41,50,51,52]. Furthermore, the levels of virus RNA in the liver of young rabbits sacrificed at five dpi are approximately 1000-fold lower than those in moribund adult rabbits [49]. Microscopically, foci of infected hepatocytes show a similar appearance to that reported in adults, and are surrounded by small infiltrates of inflammatory cells [33,41,50]. Viral antigen has also been detected in macrophages from infected kittens and adults [33,50]. In accordance with the limited liver pathology, infected kittens show only mild increases in serum transaminases compared to those observed in adult rabbits, and other hematological and biochemical parameters, such as bilirubin, blood glucose concentration, and measures of coagulation, remain unchanged [41,51]. The mechanism underlying this resistance of young rabbits is not understood, although various suggestions have been proposed. These include (i) a developmental antigen that functions as the cellular receptor for the virus [30,53]; (ii) structural or functional changes to the liver, possibly due to dietary changes at weaning, that induce susceptibility [33,50]; or (iii) differences in innate immune responses between young and adult rabbits that drive pathogenesis [51,52].

To further investigate the mechanisms underlying the innate resistance of kittens to GI.1-induced disease, we conducted a genome-wide analysis of liver transcripts from young and adult rabbits infected with GI.1, and kittens infected with GI.2. This allowed us to assess both potential virus-related factors (i.e., difference in responses between GI.1 and GI.2 infection in kittens) as well as potential age-related host factors (i.e., difference in responses between adults and kittens during GI.1 infection) contributing to the resistance observed in kittens to GI.1 RHD. Here, we discuss those genes showing strong differential regulation between the experimental groups and the corresponding pathways involved in host responses to GI.1 and GI.2 infections, and compare transcriptional responses between young and adult rabbits.

## 2. Materials and Methods

### 2.1. Experimental Treatments and Virus Inoculation

Animal experiments were conducted at the Commonwealth Scientific and Industrial Research Organisation (CSIRO) Black Mountain Laboratories following the Australian Code for the Care and Use of Animals for Scientific Purposes (2013) and approved by the CSIRO Ecosystem Sciences Animal Ethics Committee (permit identifiers: CESAEC DOMRAB, SEAEC 10-12, ESAEC 13-10). New Zealand white rabbits (36 days old or >10 weeks old) bred from GI.4 and GI.1 antibody-free does were housed either individually or in litter groups. All rabbits were confirmed negative for antibodies to the non-pathogenic calicivirus GI.4 [54] and were healthy prior to inclusion in the study. Five experimental groups were delineated: (i) six control kittens sham inoculated with phosphate-buffered saline (PBS) and with tissues harvested at 12 (*n* = 3) and 24 (*n* = 3) hpi; (ii) eight kittens infected with GI.1 and tissues harvested at 12 (*n* = 4) and 24 (*n* = 4) hpi; (iii) seven kittens infected with GI.2 and tissues harvested at 12 (*n* = 3) and 24 (*n* = 4) hpi; (iv) seven adult rabbits infected with GI.1 and tissues harvested at 12 (*n* = 3) and 24 (*n* = 4) hpi; and (v) five control adult rabbits, four of which were sham inoculated with PBS with tissues harvested at 12 (*n* = 2) and 24 (*n* = 2) hpi, as well as a retired breeding doe that was used as an additional control (Table 1). Animals were infected orally with clarified liver homogenate produced from infected rabbits diluted to either 3 × 10^8^ capsid gene copies of GI.2 or 1500 LD_50_ of GI.1 (equivalent to approximately 1.5 × 10^8^ capsid gene copies). These doses are both considered to be high infectious doses. Control animals received 1 mL of PBS. The GI.2 and GI.1 strains used for infection were the Australian field isolate BlMt-1 (GenBank #KT280060), and a commercially available titrated preparation of strain Czech-351 (GenBank #KF594475, Elizabeth Macarthur Agriculture Institute, Menangle, Australia), respectively. The Czech-351 isolate has been reported to cause a high case-fatality rate in adult domestic rabbits and a low case-fatality rate in 5 week old kittens [1,49]. A recent study reported a very high case-fatality rate of BlMt-1 in 5 week old domestic rabbits [55]. Tissues were harvested at 12 or 24 hpi, after rabbits had been anesthetised by intramuscular injection of either Zoletil 100 (Virbac, Peakhurst, NSW, Australia) or a combination of 20 mg/mL xylazine hydrochloride (Troy Laboratories, Smithfield, NSW, Australia) and 100 mg/mL ketamine hydrochloride (Mavlab, Logan, QLD, Australia), and then euthanized by intravenous or intracardiac injection of 325 mg/mL sodium pentobarbitone (Virbac). Infection times of the 12 h and 24 h groups were staggered such that all animals were sacrificed at similar times of the day to avoid any differences in gene expression caused by diurnal fluctuations in rabbit metabolism. Tissues were collected at necropsy and stored in RNAlater (Qiagen, Chadstone Centre, VIC, Australia) at −80 °C until processing.

### 2.2. RNA Extraction

Twenty to 30 mg of liver from each rabbit was homogenized with 1-mm glass beads (Daintree Scientific, St Helens, TAS, Australia) using a Precellys 24-dual tissue homogenizer (Bertin Technologies, Montigny-le-Bretonneux, France). RNA was then extracted using either the Qiagen RNeasy mini kit (Qiagen, Chadstone Centre, VIC, Australia) or the Maxwell 16 LEV simplyRNA tissue kit (Promega, Sydney, NSW, Australia) as per the manufacturers’ instructions.

### 2.3. Virus Quantification

Viral load was quantified in terms of the “capsid gene copy number” using the quantitative reverse transcription polymerase chain reaction (RT-qPCR) method that detects both GI.1 and GI.2, as described previously [22]. Briefly, reactions were performed in duplicate using the SensiFAST SYBR No-ROX One-Step kit (Bioline, Alexandra, NSW, Australia) on a BioRad CFX96/C1000 thermal cycler platform using the primers RHDV all 3 Fw 5’-TTGACRTACGCCCTGTGGGACC-3′ and RHDV all 3b Rv 5’-TCAGACATAAGARAAGCCATTRGYTG-3′ [22]. Absolute quantification was performed using a standard curve generated from in vitro generated full length GI.1 RNA transcripts of known concentration [22].

### 2.4. RNA Sequencing and Analysis

**Figure 1:**
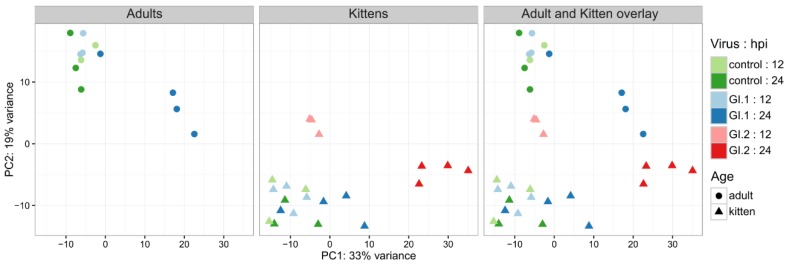
Similarity of gene expression profiles across rabbit age cohorts during infection with different GI viruses. GI.1-infected kittens had the smallest transcriptomic response after infection, while large changes were observed from 12 hpi in GI.2-infected kittens, and in GI.1-infected adults at 24 hpi. Notably, expression profiles varied considerably between adult and kitten control rabbits. Expression values from the top 500 most variable genes across the samples were normalized using regularized log transformation and plotted using principal component analysis.

Messenger RNA (mRNA) was enriched in the total RNA samples using polyA selection and sequenced using two 75 bp single-end NextSeq lanes (Illumina, Scoresby, VIC, Australia) at the Australian Cancer Research Foundation (ACRF) Biomolecular Resource Facility (The John Curtin School of Medical Research, Australian National University, Canberra, Australia). RNA-Seq reads were cleaned by removing Illumina TruSeq adapters, trimming sequences when the quality score dropped below 20, and discarding reads shorter than 50 bp using Trimmomatic v.0.32 [56]. The cleaned reads were then mapped to the rabbit genome (OryCun2.0; http://www.ensembl.org/Oryctolagus_cuniculus) and rabbit calicivirus genomes (GI.1, GenBank #KF594473; GI.2, GenBank #KT280060) using TopHat v.2.1.0 [57]. Counts for each gene in the rabbit genome were calculated using HTSeq v.0.6.0 [58] with the “union” mode for processing overlapping and multi-mapping reads. The counts were then imported into edgeR v.3.12.1 [59] for testing of significant expression differences across the treatments. The control rabbits within each age category had very similar expression profiles at both 12 and 24 h time points (Figure 1) and, therefore, these time points were combined to generate a single control pool for each age group for differential expression testing. The differentially expressed genes were tested for functional pathway enrichment using the Gene Ontology vocabulary [60] with Fisher’s exact tests in the R package topGO v.2.22.0 [61] and the KEGG framework [62] using the kegga function within limma v.3.32.1 [63]. Reads per kilobase per million (rpkm) values were calculated using the *fpkm* function in edgeR v.2.12.1 [59] and plotted with ggplot v.2.1.0 [64]. Raw coverage of the viral genomes was obtained by mapping the cleaned reads to the GI genomes using Bowtie v.2.2.9 [65], extracting the coverage profile with Samtools v.1.3.1, and plotting with Gviz v.1.14.2 [66].

Gene co-expression networks were constructed using the R package “WGCNA” (Weighted Gene Co-expression Network Analysis), following the authors’ recommendations [67]. Briefly, significantly differentially expressed genes were filtered by removing genes with low counts and log transformed. The function “pickSoftThreshold” was used to plot the data, and a soft thresholding power of 10 was chosen based on examination of the plot. The genes were clustered using the function “hclust” and the tree was cut into modules using “cutreeDynamic” with a minimum cluster size of 10. The modules were further refined using “mergeCloseModules” and manual examination of dendrograms using “plotDendroAndColors”. Co-expression modules were then tested for significant associations with the rabbit treatment groups using the R base function “cor” and plotted using “labeledHeatmap” [67].

## 3. Results

### 3.1. Genome Mapping and Significance Testing

The transcriptional response of rabbits to lagovirus infection was investigated 12 and 24 hpi in juveniles and adults, and compared to control animals for each age group. Messenger and viral RNA were enriched from the liver of each rabbit and RNA-seq was conducted, producing several million high-quality reads per sample (Table 1). More than 90% of these reads were successfully aligned to the rabbit genome, providing good coverage for the calculation of expression values (Table 1). Viral reads were only detected in rabbits at 24 hpi, indicating that viral titres at 12 hpi were too low to be detected using the sequencing depths obtained here (Table 1). Although there was considerable variation between individual animals, adults 24 h post-GI.1 infection, and kittens 24 h post-GI.2 infection, contained the highest viral loads (Table 1), which is consistent with the higher levels of virulence described for GI.1 in adult compared to young rabbits, and the recently described high case-fatality rate reported for 5 week old rabbits infected with the Australian GI.2 isolate [55]. These broad patterns were validated using RT-qPCR assays (Table 1). Viral genome copy numbers varied between individuals in each treatment group, likely representing individual variation in outbred rabbits very early during infection, although we cannot be certain that all GI.1 infected adults and all GI.2 infected kittens would have succumbed to infection. In all of the samples, viral genome coverage was consistent for approximately 5.3 kb, before a large increase in coverage at the 3’ end (Figure S1). This was likely due to the sequencing of multiple 2.1 kb subgenomic viral RNAs (co-linear with the 3’ end of the viral genome) that are produced by lagoviruses in large quantities early in infection [68]. Despite the individual variation between animals in each group, replicate rabbits within each of the treatment groups had broadly similar expression profiles, which provided high power for significance testing across the groups (Figure 1). The control samples were also very similar to each other, regardless of whether they were collected at 12 or 24 hpi (Figure 1). For this reason, the control samples at each time-point were combined into a single treatment group for the remainder of the analysis.

The number of differentially expressed genes in GI-infected animals correlated with expected disease severity (Table 2; Figure S2). For example, kittens that were resistant to disease caused by GI.1 infection (no disease/low severity), only differentially expressed 62 genes at 24 hpi. The overall transcriptional profile of this treatment group was similar to that in control animals (Figure 1). On the other hand, adult rabbits, which were susceptible to GI.1 (high severity), differentially expressed 322 genes at 24 hpi, and the transcriptional profile of infected animals at 24 hpi was generally distinct from the controls (Figure 1). In contrast to GI.1, GI.2 reached high titres in kittens (high severity; Table 1). In this case, changes in the transcriptomic profiles could be seen after only 12 hpi, and, by 24 hpi, there were 4211 dysregulated genes in the kittens, the most of any treatment group (Table 2).

### 3.2. Differential Gene Expression and Pathway Enrichment in Adults and Kittens

Several hundred genes were differentially expressed between uninfected kittens and uninfected adults (Table 2). Unsurprisingly, many of the genes upregulated in kittens compared to adults were related to growth and maturation, such as genes involved in the cell cycle, e.g., *HN1* and *CDK1*, and DNA replication, e.g., *NCAPH* (Table 3). Interestingly, the polymeric immunoglobulin receptor *PIGR* was significantly upregulated in adults compared to kittens (Table 3).

During GI.1 infection, kittens upregulated several immune related genes, including interferon induced proteins (*MX1*, *IFI44*, and *IFIT5*) and *EPCAM* (Table 4). Similarly, many of the most differentially expressed genes in GI.1-infected adults were also involved in immune processes, including *MX2*, *CD80* (B7-1), *IFIH1*, and *CXCL10* (Table 4). Functionally, both adults and kittens differentially regulated pathways such as “defense response to virus,” “negative regulation of viral genome replication,” and “immune response” (Table 5), likely reflecting the alteration of broad generic stress response pathways. At 12 hpi, an insufficient number of genes were differentially expressed to conduct pathway analyses.

### 3.3. Kittens Upregulate Important Components of Innate Immunity Compared to Adults, Which May Limit GI.1-Induced Pathology

**Figure 2:**
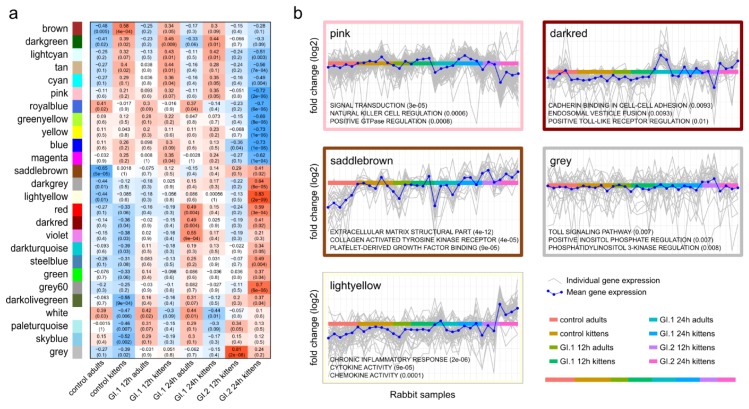
Correlation between gene co-expression modules and the rabbit treatments (**a**) and functional enrichment of select modules (**b**). The pink co-expression module contained genes that tended to be downregulated in control adults, upregulated in control kittens, further upregulated in GI.1-infected kittens, but markedly downregulated in GI.2-infected kittens. Many of the other modules were significantly correlated with the large expression changes in GI.2-infected kittens. In (**a**), the correlation coefficient is given, and the p-value is in brackets. In (**b**), significantly enriched gene ontology terms are given with the p-value in brackets.

To examine differences in the response of adults and kittens to GI infection, we used gene co-expression networks to find patterns in the data. One particular co-expression cluster (the “pink” module) contained genes that were more highly expressed by uninfected kittens compared to uninfected adults, further upregulated in GI.1-infected kittens, but markedly downregulated in GI.2-infected kittens (Figure 2; Figure S3). This pattern may suggest that the co-expressed genes were involved in the resistance of kittens to disease caused by GI.1. Functionally, the cluster was enriched for signal transduction, natural killer (NK) cell regulation, and positive GTPase regulation (Figure 2), including genes such as *HLA-DPB1*, *TNFRSF11B*, *DOK3*, and *OSGIN1* (Figure S3). Several other genes in the cluster encoded Rho-GTPases, which have a key regulatory role in immune responses [69].

**Figure 3:**
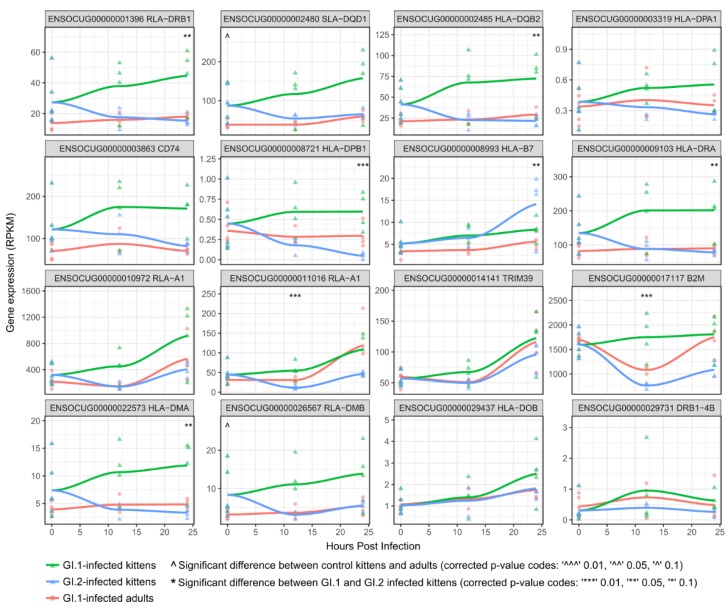
Regulation of genes in the major histocompatibility complex (MHC) during GI.1 and GI.2 infections. Several MHC genes tended to be expressed at a higher level in uninfected kittens compared to uninfected adults. Frequently, these were further upregulated in GI.1-infected kittens, but downregulated in GI.2-infected kittens (e.g., RLA-DRB1, SLA-DQD1, HLA-DQB2, CD74, HLA-DRA, HLA-DMA, and RLA-DMB). The header in each plot gives the Ensembl gene accession followed by the gene symbol.

Of particular interest was the upregulation of major histocompatibility (MHC) class II genes (e.g., *HLA-DPB1*), which are an important component of the immune system. In fact, when genes of the MHC complex were analyzed specifically, MHC class II genes tended to be expressed at a higher rate in uninfected kittens compared to adults, and their expression levels further increased in GI.1-infected kittens, beginning as early as 12 hpi (Figure 3). In contrast, the expression of many MHC II genes decreased in GI.2-infected kittens (Figure 3).

**Figure 4:**
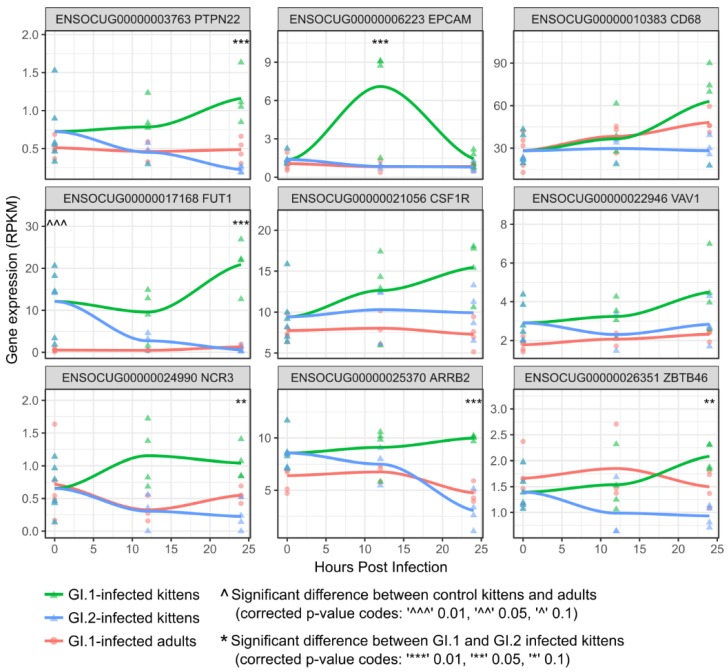
Regulation of key markers for natural killer cell (*PTPN22*, *VAV1*, *ARRB2*, and *NCR3*), macrophage (*CSF1R*, *ZBTB46*, and *CD68*), and cholangiocyte development (*EPCAM*) during GI.1 and GI.2 infections. The header in each plot gives the Ensembl gene accession followed by the gene symbol.

NK cells can express MHC class II genes, and NK cell regulation was enriched in the “pink” co-expression cluster [70]. Consequently, we specifically examined the expression of rabbit genes involved in NK cell regulation and found that several key genes, including *PTPN22*, *VAV1*, and *ARRB2*, tended to be upregulated in control kittens compared to control adults (as seen at time-point 0), and were upregulated or maintained their expression levels in GI.1-infected kittens, but were downregulated in GI.2-infected kittens (Figure 4), reflecting the co-expression pattern observed for MHC class II genes (Figure 3). Moreover, natural cytotoxicity triggering receptor 3 (*NCR3*) was also upregulated in GI.1-infected kittens compared to GI.2-infected kittens (Figure 4). Macrophages also contribute to a higher expression of MHC class II genes [71]. Thus, we examined the expression of rabbit genes likely to be associated with macrophage function, and found that a number of these genes, including *CSF1R*, *ZBTB46*, and *CD68*, tended to be upregulated by 24 hpi (if not earlier) in GI.1-infected kittens compared with GI.1-infected adults and GI.2-infected kittens (Figure 4). Another type of cell that may have contributed to MHC class II expression in GI.1-infected kittens are cholangiocytes (biliary epithelial cells). These cells not only express MHC class II genes, but also many other genes that were upregulated in GI.1-infected kittens, such as genes encoding *MX* proteins, interferons and adhesion molecules [72] (Table 4 and Table 5). In fact, *EPCAM*, which is a biomarker of cholangiocytes and their progenitor cells [72,73], was the most up-regulated gene at 12 hpi in GI.1-infected kittens (Table 4; Figure 4). In addition, the gene encoding *FUT1*, an enzyme belonging to the family of alpha 1,2 fucosyltransferases, was found to be upregulated in control kittens compared to adults and further upregulated in GI.1-infected kittens, but downregulated in GI.2-infected kittens (Figure 4).

**Figure 5:**
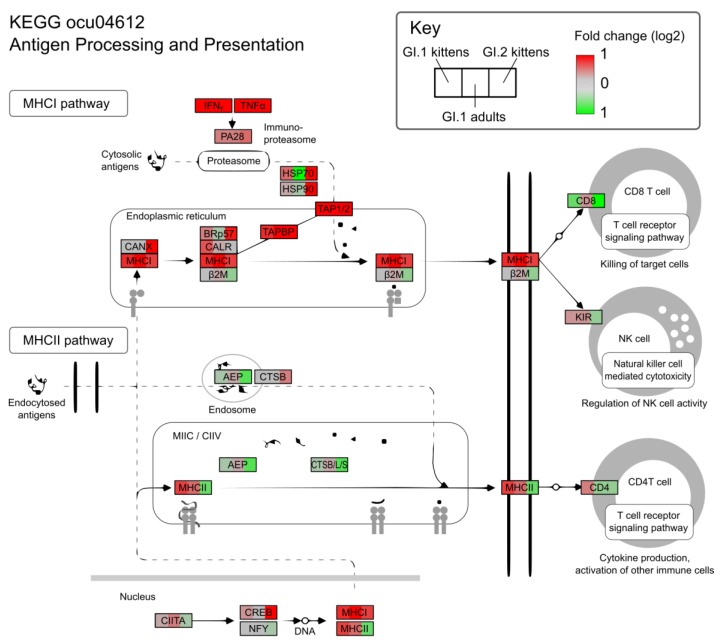
Regulation of antigen processing and presentation genes during GI.1 and GI.2 infections. GI.1-infected kittens upregulated pathways involved in natural killer cell and CD4 T-cell activation, such as MHC class II genes, while the same pathways were downregulated in GI.2-infected kittens. GI.1-infected adults also upregulated MHC class II genes, but to a lesser degree.

In the context of the KEGG pathway “Antigen Processing and Presentation”, the up-regulation of MHC class I and II genes in GI.1-infected kittens coincided with increased expression of markers associated with NK cells, such as *KLRD1* and *NKG2-A*/*NKG2-B* type II integral membrane protein, and CD4 T-cells, including the T-cell surface glycoprotein, *CD4* (Figure 5). Adults infected with GI.1 also tended to slightly up-regulate MHC genes, and these were associated with minor increases in NK cell markers, however, the magnitude was much less than for kittens (Figure 3 and Figure 5).

### 3.4. In Contrast to GI.1, GI.2 Infection Restricts the Activation of Several Innate Immune Pathways

GI.2-infected kittens differentially expressed far more genes than any other treatment group (Table 2; Figure S2). Similar to other experimental groups, however, GI.2-infected kittens upregulated generic viral response pathways involved in the negative regulation of viral genome replication, viral defense responses, and more general immune and inflammatory responses (Tables S1 and S2). A number of genes involved in immune responses were amongst the most up-regulated in GI.2-infected kittens, including *FLVCR1*, *IRAK2*, and the cell adhesion molecules *VCAM1* and *ICAM1* (Table S1). However, many of the innate immunity genes that were upregulated in GI.1-infected kittens were downregulated in GI.2-infected kittens (Figure 3, Figure 4 and Figure 5).

The downregulation of genes encoding MHC complex molecules in GI.2-infected kittens coincided with decreased expression of markers associated with CD8 T cells, NK cells, macrophages, and CD4 T cells (Figure 4 and Figure 5).

## 4. Discussion

We undertook a genome-wide transcriptome study to investigate differences in the immune responses of young and adult rabbits during very early stages of infection with two caliciviruses, GI.1 and GI.2. Interestingly, while adults are susceptible to GI.1, young rabbits are resistant [1]. In contrast, both age groups are susceptible to fulminant disease caused by GI.2 [2,5].

Several components of the innate immune system were identified as potentially important in the resistance of kittens to GI.1, including MHC class II genes. These genes were constitutively expressed at a higher rate in uninfected kittens compared to uninfected adults, suggesting that kittens have a “primed” innate immune system. Upon infection of kittens with GI.1, a further upregulation of MHC class II alleles was seen from as early as 12 hpi, indicating a rapid and coordinated innate immune response. The precise nature of this immune response, however, depends on the type of cell expressing the MHC II molecules. For example, MHC class II molecules are cell surface proteins generally expressed by antigen presenting cells (APCs), such as macrophages, natural killer (NK) cells and B cells [70,74]. In this study, macrophages and NK cells were likely to be the primary MHC II-producing cells due to the early sampling times, which precluded the development of B cell populations specific to viral antigens. We also found several markers suggesting that cholangiocytes, which are also MHC II-producing cells and potentially APCs [72,75], may play an important role in the innate resistance of kittens to GI-induced disease. The upregulation of MHC II genes indicates that either individual APCs expressed more of these molecules, or alternatively, that a greater number of APCs were present. Although high throughput sequencing cannot discriminate between these possibilities, a combination of the two processes is likely. In either case, our findings suggest that the increased activity of NK cells, macrophages, and/or cholangiocytes, have a critical role in limiting GI.1-induced pathology in young rabbits.

NK cells provide front-line innate immunity and are well known for controlling viral spread. Indeed, individuals with lower NK cell numbers or reduced NK cell-mediated cytotoxicity are far more susceptible to certain viral infections [76,77]. Here, in addition to higher MHC II expression, we found several other indications that NK cells play an important role in the resistance of kittens to GI.1. For example, a co-expressed cluster of genes containing several involved in NK cell activity, were highly expressed in GI.1-infected kittens compared to GI.1-infected adults and GI.2-infected kittens. Genes in this cluster included natural cytotoxicity triggering receptor 3 (*NCR3* or *NKp30*), which is a specialized receptor on the surface of NK cells that plays a key role in the recognition and destruction of virus-infected cells [78]. Several other potentially important genes in this cluster may be expressed as part of the same process, including *PTPN22*, *VAV1*, and *EPCAM*. For example, *PTPN22* encodes lymphoid protein tyrosine phosphatase, a protein expressed only in immune cells, and most highly expressed by NK cells [79,80]. *PTPN22* also induces cytotoxic activity of NK cells through the activation of an array of proteins including the guanine nucleotide-exchange factor, *VAV1* [81]. *VAV1* facilitates the reorganization of actin cytoskeletal proteins and augments the expression of cell adhesion molecules on cell surfaces [81,82]. In line with this, many of the most differentially expressed genes in infected rabbits were cell adhesion molecules, including *EPCAM* (epithelial cell adhesion molecule). In fact, *EPCAM* was the most differentially expressed gene in GI.1-infected kittens at 12 hpi, correlating with increases in *VAV1* expression. Moreover, a number of different GTPases were part of the co-expressed gene cluster, which may be used to boost NK cell cytotoxicity [82]. Overall, the coordinated expression of these genes suggests an orchestrated effort to increase the activity and cytotoxicity of NK cells in kittens and points to an important role for NK cells in GI.1-resistant kittens.

Macrophages may also play a role in the resistance of kittens to disease caused by GI.1. Indeed, a greater abundance of macrophages in the liver of control kittens compared to control adults, with further increases in kittens after GI.1 infection, could account for the MHC II expression patterns that we observed. Moreover, the expression of *CSF1R* (macrophage colony-stimulating factor 1 receptor) was higher in uninfected kittens compared to uninfected adults, and steadily increased over the GI.1 infection time-points, but remained stable in GI.2-infected kittens. *CSF1R* is a transmembrane protein that is almost ubiquitously expressed on mononuclear phagocytes, and can be used to differentiate macrophages from most dendritic-cell types [83,84]. We also found that the “classical” macrophage marker, *CD68* [85], and a more recently recognized marker, *ZBTB46* [86], tended to be most highly expressed in GI.1-infected kittens at 24 hpi, further suggesting a role for macrophages in clearing GI.1 virus from kittens.

In the present study, there were several indications that cholangiocytes might also be important in the resistance of kittens to GI.1-induced disease. Cholangiocytes can express MHC II molecules and comprise a relatively large proportion of cell mass in the liver (4–5%) [72]. Traditionally, the immunological contribution of these cells was thought to be restricted to immunoglobulin secretion. More recently, however, cholangiocytes have been implicated in many innate and adaptive immune processes, such as the recognition of pathogen-associated molecular patterns (PAMPs), secretion of cytokines and antimicrobial peptides, and interactions with other immune cells through the expression of cell-surface adhesion molecules (for a review, see Reference [72]). *EPCAM*, which was highly expressed in GI.1-infected kittens at 12 hpi, is frequently used as a biomarker of cholangiocytes [72,73], and *EPCAM* receptors are present on immune modulatory cells such as lymphocytes, monocytes, dendritic cells, and NK cells [87]. Moreover, cholangiocytes express *MX* proteins [72], and *MX1* was one of the most upregulated genes in GI.1-infected kittens at 24 hpi, along with several interferons. *MX* proteins, which are induced by interferons, have broad antiviral activity against RNA viruses through the recognition of viral nucleocapsid proteins and subsequent inhibition of viral replication [88]. Although antiviral activity of *MX* proteins has never been demonstrated for GI viruses, it is tempting to speculate that the upregulation of *MX-1* constitutes a possible mechanism contributing to the observed reduction of GI.1 replication in kittens. Cholangiocytes may also be associated with other immune responses, such as the observed upregulation of macrophage markers. For instance, cholangiocytes can secrete molecules that attract monocytes and macrophages during liver disease [72,89].

Of note was the differential expression of *FUT1*, which encodes an enzyme belonging to the family of alpha 1,2 fucosyltransferases. These enzymes are required for the synthesis of the histo-blood group antigen (HGBA) H type 2, which has been described as a co-receptor for lagovirus binding [28]. HBGA H type 2 is present on mucosal surfaces, such as epithelial cells of the trachea and duodenum, but is not expressed in liver parenchyma [29,30]. *FUT1* expression levels were elevated in kittens compared to adults, upregulated in GI.1-infected kittens at 24 hpi, but downregulated in GI.2-infected kittens as early as 12 hpi. This could suggest that differential fucosylation patterns of immune cell ligands and receptors may contribute to the attenuated GI-1 infections in kittens.

We propose that young kittens have a primed innate immune system that perhaps compensates for the lack of a fully developed adaptive immune system. Possible causes for elevated innate immune responses in rabbit kittens are unclear but warrant further investigations. Exposure to a suite of different antigens while the rabbit’s complex gut microbiome is establishing after birth is a possibility. It is also of note that the waning of age-related resistance to lethal GI.1 infection coincides with weaning and a change from a high-fat diet to a vegetarian diet. Links between high-fat diets and inflammation have been demonstrated in adult rabbits used as a model for human diabetes [90], although no such links have yet been reported in very young rabbits.

The consequences of this primed innate immunity includes coordinated increases in the expression of genes associated with NK cell, macrophage, and cholangiocyte activity, which may allow kittens to rapidly respond to and limit infection with GI.1. Indeed, previous studies support this assertion. First, treatment of adult rabbits with poly(I:C) to artificially “prime” the innate immune system by stimulating type 1 interferon responses can protect against GI.1 challenge for up to six hours post-treatment [91]. Second, adult rabbits treated with the pro-inflammatory cytokine cardiotrophin-1, a member of the IL-6 cytokine family, prior to infection with GI.1 had lower case fatality rates, improved biochemical parameters of liver function, and less severe hepatic necrosis based on histopathology, although virus loads in the liver reached similar titres to untreated rabbits [92]. Thirdly, GI.1 infection of kittens was associated with increased levels of circulating pro-inflammatory cytokines as early as 6 hpi, along with a rapid influx of macrophages and lymphocytes into the liver, although these results were not compared to adult rabbits [4]. Perhaps most convincingly, immunosuppression of young rabbits with corticosteroid treatment negated their innate resistance to GI.1-induced disease, with kittens developing widespread hepatic necrosis indistinguishable from that of adult animals and succumbing to disease in 24 to 72 hpi [52]. It should be noted, however, that corticosteroids also have wide-ranging systemic effects on multiple metabolic pathways, and treatment may lead to functional changes in the liver that could increase susceptibility to infection. This innate resistance of young rabbits to GI.1-induced disease is not observed with GI.2; indeed, mortalities have been observed in rabbit kittens as young as 11 days old [93].

Compared to kittens infected with GI.1, GI.2-infected kittens had decreased expression of several MHC class I genes, including those encoding the MHC class I structural protein, *B2M* (beta-2-microglobulin). *B2M* forms part of the alpha chain of the MHC class I complex, and is required for the assembly and cell surface expression of functional MHC class I molecules [74]. The limited expression of these pathways during GI.2 infection suggests that GI.2 may suppress host innate immune responses, allowing the virus to replicate rapidly. Indeed, there were more viral genome copies at 24 hpi in GI.2-infected kittens compared to any other treatment group (Table 1). Some viruses intentionally downregulate host MHC class I molecules to avoid recognition by CD8 T cells [94]. The consequence of this downregulation, however, is that the virus-infected cells become susceptible to NK cell attack [95]. In turn, certain viruses have evolved the ability to preferentially express, or to mimic the expression of, ligands that inhibit NK cell receptors [96]. While these examples involve large double-stranded DNA viruses, small RNA viruses have also evolved mechanisms to avoid host immune responses. For example, murine norovirus (MNV), which belongs to the same family as lagoviruses (*Caliciviridae*), expresses a protein that delays the upregulation of genes involved in host innate immune responses [97]. Thus, although host immune modulation by GI.2 has not been demonstrated, caliciviruses as a family are known to subvert host responses, and the functions of several of the non-structural proteins of the lagoviruses are as yet unknown [98]. Moreover, variability in MHC class I genes has been implicated in the genetic resistance of rabbits to GI.1 viruses [99]. It is feasible that when genetic resistance against GI.2 begins to develop in wild rabbit populations, the mechanism may be different. These examples provide some suggestion that GI.2 may have the ability to modulate the expression of MHC class I molecules, and enable the virus to replicate and spread before the development of an immune response. This scenario should be tested in future studies.

## 5. Conclusions

Overall, we found that kittens infected with GI.1 increased the expression of multiple genes encoding components of the innate immune response compared with adult rabbits, particularly those associated with MHC II genes, such as natural killer cells, macrophages, and cholangiocytes. In contrast, these genes were downregulated in kittens during GI.2 infection, suggesting that these genes, including MHC class II alleles and those involved in NK cell regulation, play a role in the pathogenicity differences of GI.1 and GI.2. We propose a model where young kittens have a primed innate immune system, perhaps due to the increased load of environmental antigens they encounter at this age, or due to a high-fat milk diet stimulating innate immune responses. This primed innate immunity allows them to respond rapidly to the incoming viral pathogen. These innate responses may limit GI.1 replication to a point where only individual hepatocytes become infected, precluding progression to the coalescing hepatitis seen in adult animals. In contrast, GI.2 may be able to suppress these innate responses sufficiently to permit extensive viral replication, leading to the fulminant hepatitis observed in GI.2-infected kittens, although the mechanism by which GI.2 induces this suppression requires further elucidation. This model is supported by our data and earlier work done by others demonstrating the importance of a robust immune system in the natural resistance of young rabbits to GI.1-induced disease [52]. However, these findings need to be validated experimentally in the future, for example, by demonstrating changes in expression levels of key proteins implicated in this study. The fundamental host and viral mechanisms underlying the resistance of young rabbits to disease induced by GI.1, but not GI.2, also has important epidemiological implications. The ability of GI.2 to infect new cohorts of rabbits at a much younger age likely represents a key competitive advantage over GI.1. Our findings therefore represent an important step in increasing our understanding of how an emerging pathogen (i.e., GI.2) can displace a highly successful existing pathogen (GI.1) in a widely dispersed host population. This may ultimately lead to improved management of wild rabbit populations, either to better protect domestic and wild rabbits in their native range, or to improve the management of overabundant rabbit populations in parts of the world where these are considered an invasive pest.

## Figures and Tables

**Table 1:** Experimental design and RNA-Seq mapping results.

ID	Virus	hpi	Age	Total Reads	Cleaned Reads	Mapped to Rabbit Genome *	Mapped to GI Genome ^	RT-qPCR (Copies/mg Tissue)
A130	cont	N/A **	A	36,608,811	33,019,057	30,372,424 (92.0%)	0 (0.00%)	55
A440	cont	12	A	31,544,539	29,690,688	27,851,164 (93.8%)	0 (0.00%)	0
A445	cont	12	A	33,024,742	30,839,504	28,557,666 (92.6%)	0 (0.00%)	0
K11	cont	12	K	30,376,750	28,436,074	26,697,303 (93.9%)	0 (0.00%)	0
K2	cont	12	K	26,466,336	24,621,221	23,121,670 (93.9%)	0 (0.00%)	0
K9	cont	12	K	38,879,760	35,838,449	33,677,252 (94.0%)	0 (0.00%)	0
A446	cont	24	A	26,875,056	25,190,314	23,565,537 (93.5%)	0 (0.00%)	0
A447	cont	24	A	30,801,403	28,647,130	26,648,792 (93.0%)	0 (0.00%)	0
K12	cont	24	K	21,522,682	19,510,404	18,264,437 (93.6%)	0 (0.00%)	0
K14	cont	24	K	32,273,375	30,146,422	28,183,027 (93.5%)	0 (0.00%)	0
K3	cont	24	K	34,270,680	31,856,522	29,771,558 (93.5%)	0 (0.00%)	0
A444	GI.1	12	A	41,554,574	38,521,840	35,819,416 (93.0%)	0 (0.00%)	94
A451	GI.1	12	A	34,269,029	31,413,217	29,237,190 (93.1%)	0 (0.00%)	28
A452	GI.1	12	A	30,744,544	28,741,202	26,469,612 (92.1%)	0 (0.00%)	0
K1	GI.1	12	K	35,648,932	33,188,895	31,053,518 (93.6%)	0 (0.00%)	0
K13	GI.1	12	K	28,834,630	26,852,299	25,283,903 (94.2%)	0 (0.00%)	0
K5	GI.1	12	K	32,247,351	30,065,559	28,089,931 (93.4%)	0 (0.00%)	0
K7	GI.1	12	K	34,983,905	32,474,265	30,104,239 (92.7%)	0 (0.00%)	85
A443	GI.1	24	A	32,025,317	29,266,843	27,437,173 (93.7%)	4321 (0.01%)	1,359,870
A448	GI.1	24	A	41,956,002	38,817,349	35,919,297 (92.5%)	817 (0.00%)	213,792
A449	GI.1	24	A	32,829,766	29,594,927	27,422,586 (92.7%)	352 (0.00%)	83,234
A450	GI.1	24	A	39,743,588	37,093,310	34,584,907 (93.2%)	5 (0.00%)	1205
K10	GI.1	24	K	26,707,074	24,485,698	22,779,842 (93.0%)	2 (0.00%)	307
K4	GI.1	24	K	35,102,440	32,387,798	30,179,957 (93.2%)	49 (0.00%)	18,929
K6	GI.1	24	K	30,314,748	27,870,816	25,865,930 (92.8%)	19 (0.00%)	6405
K8	GI.1	24	K	30,187,241	28,311,539	26,206,586 (92.6%)	5 (0.00%)	1490
K379	GI.2	12	K	32,179,571	30,150,297	28,485,814 (94.5%)	0 (0.00%)	81
K380	GI.2	12	K	30,937,894	29,113,196	27,110,731 (93.1%)	0 (0.00%)	66
K381	GI.2	12	K	24,208,541	22,521,030	20,774,527 (92.2%)	0 (0.00%)	0
K375	GI.2	24	K	31,299,465	29,544,132	27,913,303 (94.5%)	26,558 (0.09%)	20,966,939
K376	GI.2	24	K	30,626,983	28,656,602	26,667,507 (93.1%)	1410 (0.00%)	3,634,306
K377	GI.2	24	K	35,624,017	33,340,447	31,470,468 (94.4%)	1947 (0.00%)	323,906
K378	GI.2	24	K	26,510,091	24,505,534	22,803,925 (93.1%)	22,528 (0.09%)	4,857,896

* rabbit genome is OryCun2.0 (http://www.ensembl.org/Oryctolagus_cuniculus). ^ GI.1 is GenBank#KF594473, and GI.2 is GenBank #KT280060. ** A130 was an additional negative control animal that was not part of the infection experiment. A = adult, k = kitten, cont = noninfected control, hpi = hours post infection.

**Table 2:** Number of significantly differentially expressed genes (false discovery rate < 0.05).

Comparison	Upregulated	Downregulated
Control adults vs. control kittens	556 (up in adults)	817 (up in kittens)
GI.1-infected kittens (12 hpi) *	2	1
GI.1-infected kittens (24 hpi) *	60	2
GI.1-infected adults (12 hpi) *	2	0
GI.1-infected adults (24 hpi) *	296	26
GI.2-infected kittens (12 hpi) *	347	293
GI.2-infected kittens (24 hpi) *	2152	2059

* Compared to control animals.

**Table 3:** The top 10 most differentially expressed genes between control adults and control kittens.

Ensembl Gene #	Annotation	Log Fold Change ^	FDR *
ENSOCUG00000005528	*HAL* (histidine ammonia-lyase)	2.9	3.69 × 10^−19^
ENSOCUG00000014137	*NAALAD2* (N-acetylated alpha-linked acidic dipeptidase 2)	−3.8	4.71 × 10^−16^
ENSOCUG00000002950	*HN1* (hematological and neurological expressed 1)	−1.9	1.99 × 10^−15^
ENSOCUG00000003663	*ACTG2* (actin, gamma 2, smooth muscle, enteric)	−2.3	7.09 × 10^−13^
ENSOCUG00000014725	N/A	6.9	8.92 × 10^−13^
ENSOCUG00000014317	*PIGR* (polymeric immunoglobulin receptor)	2.3	1.70 × 10^−11^
ENSOCUG00000023455	N/A	−3.0	1.70 × 10^−11^
ENSOCUG00000002858	*CDK1* (cyclin dependent kinase 1)	−4.7	2.35 × 10^−11^
ENSOCUG00000009882	*NCAPH* (condensing complex subunit 2)	−2.9	2.35 × 10^−11^
ENSOCUG00000012902	N/A	−4.0	2.35 × 10^−11^

* FDR is the false discovery rate. ^ Positive fold change was upregulated in adults. N/A indicates unannotated gene.

**Table 4:** The top differentially expressed genes in GI.1-infected rabbits.

Ensembl Gene #	Annotation	Log Fold Change	FDR *
*Adults after 12 h*			
ENSOCUG00000023455	N/A	2.4	0.0003
ENSOCUG00000025530	N/A	2.3	0.0350
*Adults after 24 h*			
ENSOCUG00000026233	*HRASLS2* (HRAS like suppressor 2)	7.4	8.32 × 10^−13^
ENSOCUG00000029154	N/A	5.4	3.30 × 10^−12^
ENSOCUG00000002863	*IFIH1* (interferon induced with helicase C domain 1)	3.4	1.31 × 10^−11^
ENSOCUG00000009504	*CD80* (T-lymphocyte activation antigen CD80)	3.5	1.31 × 10^−11^
ENSOCUG00000020931	*SAMD9* (sterile alpha motif domain containing 9)	4.6	5.12 × 10^−11^
ENSOCUG00000021037	*MX2* (MX dynamin like GTPase 2)	5.4	1.81 × 10^−10^
ENSOCUG00000010311	*NXPE4* (neurexophilin and PC-esterase domain family member 4)	3.7	3.05 × 10^−10^
ENSOCUG00000009811	*ZNFX1* (zinc finger NFX1-type containing 1)	2.6	3.61 × 10^−10^
ENSOCUG00000000716	*EPSTI1* (epithelial stromal interaction 1)	4.7	5.53 × 10^−10^
ENSOCUG00000016280	*CXCL10* (C-X-C motif chemokine ligand 10)	6.8	5.53 × 10^−10^
*Kittens after 12 h*			
ENSOCUG00000006223	*EPCAM* (epithelial cell adhesion molecule)	2.3	2.84 × 10^−5^
ENSOCUG00000009559	*KIAA1841*	1.3	0.0472
ENSOCUG00000011988	N/A	−3.8	0.0472
*Kittens after 24 h*			
ENSOCUG00000004501	N/A	4.1	4.62 × 10^−6^
ENSOCUG00000006595	*UBA7* (ubiquitin like modifier activating enzyme 7)	2.9	1.32 × 10^−5^
ENSOCUG00000021924	*MX1* (MX dynamin like GTPase 1)	4.2	1.84 × 10^−5^
ENSOCUG00000006482	*IFI44* (interferon induced protein 44)	2.9	0.0001
ENSOCUG00000015823	*OAS2* (2’-5’-oligoadenylate synthetase 2)	3.3	0.0001
ENSOCUG00000013278	*DHX58* (DExH-box helicase 58)	2.5	0.0002
ENSOCUG00000027981	*ISG15* (ubiquitin-like modifier)	5.4	0.0002
ENSOCUG00000015872	*HERC5* (HECT and RLD domain containing E3 ubiquitin protein ligase 5)	2.6	0.0002
ENSOCUG00000024570	*IFIT5* (interferon induced protein with tetratricopeptide repeats 5)	2.6	0.0002
ENSOCUG00000024734	N/A	2.7	0.0002

A maximum of ten differentially expressed genes is shown per experimental group. * FDR is the false discovery rate. N/A indicates unannotated genes.

**Table 5:** Significantly enriched Gene Ontology (GO) terms in the category “Biological Process” for upregulated genes in GI.1-infected rabbits.

GO #	Term	Annotated	Significant	Expected	*p*-Value
*Adults after 24 h*					
GO:0051607	defense response to virus	106	29	1.72	2.40 × 10^−19^
GO:0045071	negative regulation of viral genome replication	27	12	0.44	3.50 × 10^−15^
GO:0006955	immune response	672	70	10.92	5.70 × 10^−13^
GO:0071360	cellular response to exogenous dsRNA	11	5	0.18	4.60 × 10^−7^
GO:0035455	response to interferon-alpha	12	5	0.19	7.80 × 10^−7^
GO:0002474	antigen processing and presentation of peptide antigen via MHC class 1	12	5	0.19	7.80 × 10^−7^
GO:0060333	interferon-gamma-mediated signaling pathway	12	5	0.19	7.80 × 10^−7^
GO:0034123	positive regulation of toll-like receptor signaling pathway	13	5	0.21	1.30 × 10^−6^
GO:0050688	regulation of defense response to virus	38	9	0.62	2.10 × 10^−6^
*Kittens after 24 h*					
GO:0051607	defense response to virus	106	17	0.37	4.60 × 10^−17^
GO:0045071	negative regulation of viral genome replication	27	9	0.09	1.30 × 10^−16^
GO:0035455	response to interferon-alpha	12	6	0.04	1.10 × 10^−12^
GO:0032727	positive regulation of interferon-alpha production	11	4	0.04	4.00 × 10^−8^
GO:0032728	positive regulation of interferon-beta production	22	4	0.08	8.60 × 10^−7^
GO:0009615	response to virus	155	21	0.53	2.00 × 10^−6^
GO:0039529	RIG-I signaling pathway	10	3	0.03	4.50 × 10^−6^
GO:0006955	immune response	672	21	2.31	2.60 × 10^−5^
GO:0035456	response to interferon-beta	14	3	0.05	6.60 × 10^−5^
GO:0019941	modification-dependent protein catabolic process	354	4	1.22	0.00011

## Data Availability

Raw RNA-seq reads have been deposited in the NCBI Sequence Read Archive under BioProject accession PRJNA434149. A javascript app is also publically available for the exploration and visualisation of this dataset (https://neavemj.github.io/posts/D3_rab_app).

## Supplementary Materials

The following are available online at http://www.mdpi.com/1999-4915/10/9/512/s1, Figure S1: Read coverage of the GI.1 and GI.2 genomes, Figure S2: Significantly differentially expressed genes in infected rabbits compared to controls. Figure S3: Expression of genes in the “pink” co-expression module and change in selected genes over time. Table S1: The top 10 most differentially expressed genes in GI.2-infected rabbits. Table S2: Significantly enriched Gene Ontology (GO) terms in the category “Biological Process” for up regulated genes in GI.2-infected rabbits.
