# Supplementary material for: Robust Innate Immunity of Young Rabbits Mediates Resistance to Rabbit Hemorrhagic Disease Caused by Lagovirus Europaeus GI.1 But Not GI.2
Source: Viruses. 2018 Sep 19;10(9):512. doi: 10.3390/v10090512 (PMC6163550; doi:10.3390/v10090512)
Supplement: Supplementary file 1 [file viruses-10-00512-s001.pdf]

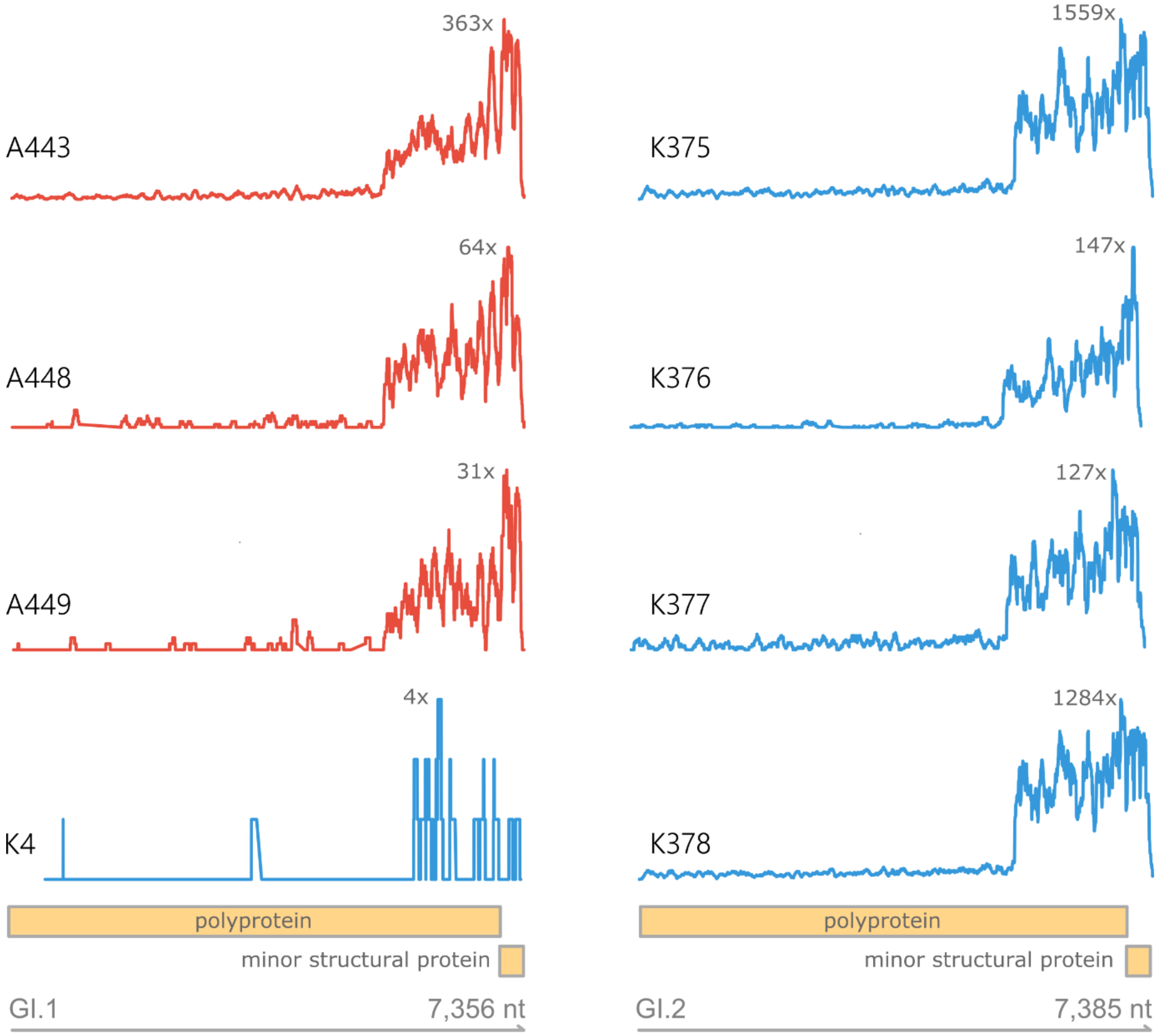

Supplementary Figure 1. Read coverage of the GI.1 and GI.2 genomes. The left panel shows viral genome coverage in rabbits infected with GI.1 and the right with GI.2. Red lines indicate adults, blue lines indicate kittens, and the highest coverage peak for each sample is annotated in grey text. Only samples with more than 20 reads mapping to the viral genomes are shown (see Table 1).

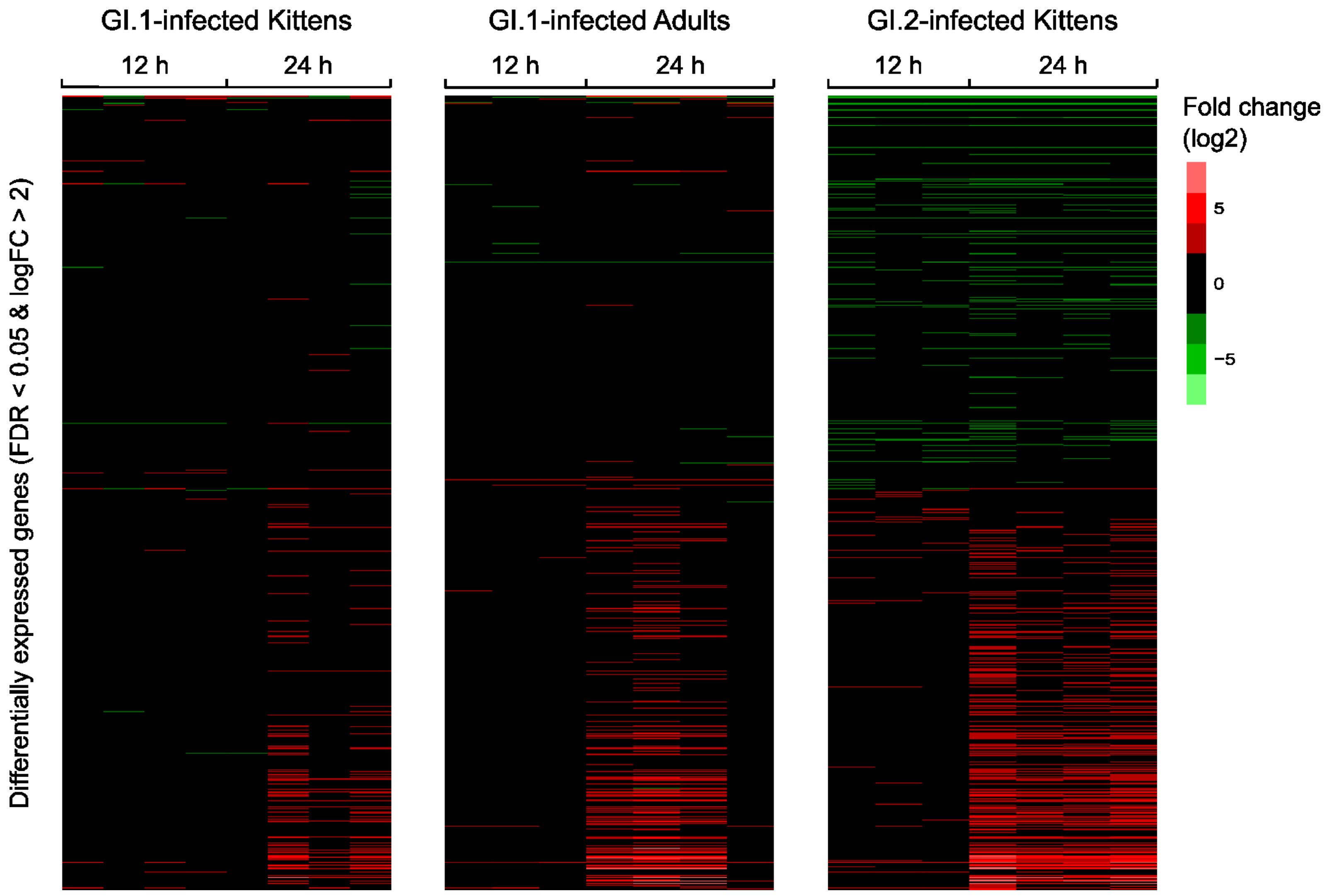

Supplementary Figure 2. Significantly differentially expressed genes in infected rabbits compared to controls. Kittens infected with GI.2 showed a greater transcriptional response compared to the other treatment groups, evidenced by more differentially expressed genes. In particular, more genes were down-regulated and changes were clearly detectable by 12 hours post-infection. FDR is “false discovery rate” and FC is “fold change”.

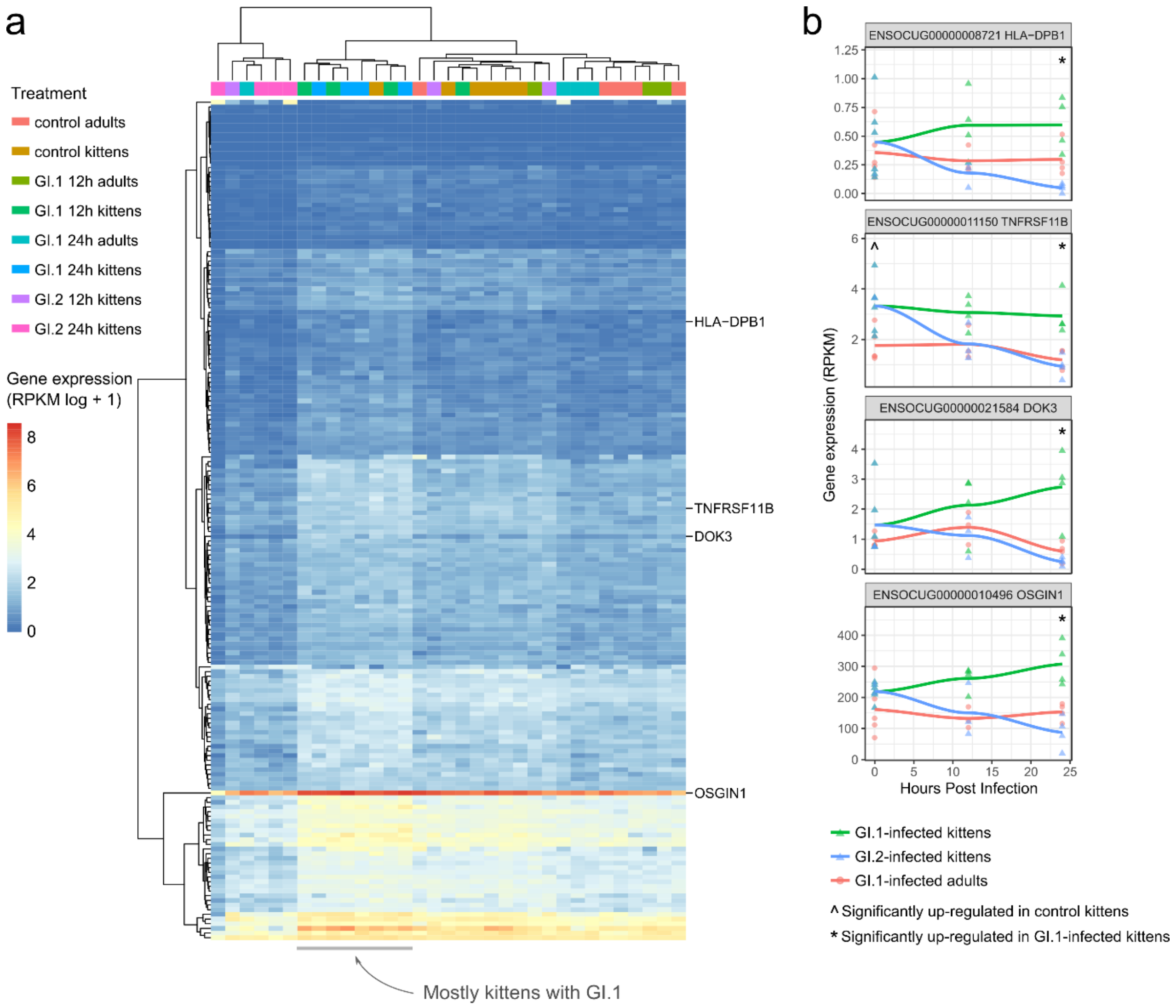

Supplementary Figure 3. Expression of genes in the "pink" co-expression module (a) and change in selected genes over time (b). Genes in this co-expression cluster tended to be up-regulated in control kittens, further up-regulated in GI.1-infected kittens but down-regulated in GI.2-infected kittens, suggesting a role in the resistance of kittens to GI.1. Gene expression was normalised using reads per kilobase per million (RPKM). In (b) the header in each plot gives the Ensembl gene accession followed by the gene symbol.

Supplementary Table S1. The top 10 most differentially expressed genes in GI.2-infected rabbits.

Ensembl gene #	Annotation	Log fold change	FDR*
Kittens after 12 hours			
ENSOCUG00000024623	N/A	-6.2	1.04E-15
ENSOCUG00000002051	N/A	-8.0	3.52E-11
ENSOCUG00000025827	*NAT8* (N-acetyltransferase 8 (putative))	-4.1	2.83E-10
ENSOCUG00000020913	N/A	6.1	9.03E-10
ENSOCUG00000028214	N/A	-7.6	7.96E-09
ENSOCUG00000012529	N/A	1.9	1.28E-08
ENSOCUG00000029158	N/A	-5.0	1.28E-08
ENSOCUG00000024402	N/A	2.6	2.25E-08
ENSOCUG00000003017	*ERICH3* (glutamate rich 3)	3.6	2.59E-08
ENSOCUG00000000308	N/A	-8.6	2.80E-08
Kittens after 24 hours			
ENSOCUG00000011697	*VCAM1* (vascular cell adhesion molecule 1)	2.7	4.00E-30
ENSOCUG00000000023	*PKP2* (plakophilin 2)	2.5	1.60E-24
ENSOCUG00000024623	N/A	-6.5	2.46E-22
ENSOCUG00000025827	*NAT8* (N-acetyltransferase 8 (putative))	-5.3	1.96E-21
ENSOCUG00000026273	N/A	2.7	2.42E-21
ENSOCUG00000000128	*C21orf91* (chromosome 21 open reading frame 91)	3.8	2.53E-21
ENSOCUG00000004804	*FLVCR1* (feline leukemia virus subgroup C cellular receptor 1)	3.4	4.60E-20
ENSOCUG00000029644	*ICAM1* (intercellular adhesion molecule 1)	3.1	8.08E-20
ENSOCUG00000002819	*IRAK2* (interleukin 1 receptor associated kinase 2)	2.3	1.70E-19
ENSOCUG00000007089	*PLK2* (polo like kinase 2)	1.8	3.09E-19

* FDR is the False Discovery Rate

N/A indicates unannotated genes

Supplementary Table S2. Significantly enriched Gene Ontology (GO) terms in the category "Biological Process" for up regulated genes in GI.2-infected rabbits.

GO #	Term	Annotated	Significant	Expected	p-value
Kittens after 12 hours					
GO:0005975	carbohydrate metabolic process	515	23	9.76	2.60E-08
GO:0051968	positive regulation of synaptic transmission, glutamatergic	10	4	0.19	2.40E-05
GO:0045332	phospholipid translocation	21	5	0.4	3.70E-05
GO:0042493	response to drug	104	10	1.97	0.00021
GO:0008152	metabolic process	8367	171	158.52	0.00054
GO:0071625	vocalization behavior	10	3	0.19	0.00073
GO:0019318	hexose metabolic process	117	7	2.22	0.00114
GO:0060291	long-term synaptic potentiation	28	4	0.53	0.0018
GO:0042886	amide transport	175	9	3.32	0.0025
GO:0034446	substrate adhesion-dependent cell spreading	60	5	1.14	0.00263
Kittens after 24 hours					
GO:0045071	negative regulation of viral genome replication	27	20	3.23	1.20E-13
GO:0071260	cellular response to mechanical stimulus	34	19	4.07	8.50E-10
GO:0051607	defense response to virus	106	46	12.68	2.80E-09
GO:0045944	positive regulation of transcription from RNA polymerase II promoter	710	144	84.91	6.10E-09
GO:0050729	positive regulation of inflammatory response	55	22	6.58	1.20E-08
GO:0006955	immune response	672	190	80.36	1.30E-08
GO:0071222	cellular response to lipopolysaccharide	98	38	11.72	4.30E-08
GO:0043123	positive regulation of I-kappaB kinase/NF-kappaB signaling	123	38	14.71	1.90E-07
GO:0035455	response to interferon-alpha	12	9	1.44	7.70E-07
GO:0001961	positive regulation of cytokine-mediated signalling pathway	18	11	2.15	9.90E-07
